# Depression, obesity and their comorbidity during pregnancy: effects on the offspring’s mental and physical health

**DOI:** 10.1038/s41380-020-0813-6

**Published:** 2020-07-06

**Authors:** Nadia Cattane, Katri Räikkönen, Roberta Anniverno, Claudio Mencacci, Marco A. Riva, Carmine M. Pariante, Annamaria Cattaneo

**Affiliations:** 1 Biological Psychiatry Unit, IRCCS Istituto Centro San Giovanni di Dio Fatebenefratelli, Brescia, Italy; 2 Department of Psychology and Logopedics, Faculty of Medicine, University of Helsinki, Helsinki, Finland; 3 Department of Neuroscience, ASST Fatebenefratelli Sacco, Milan, Italy; 4 Department of Pharmacological and Biomolecular Sciences, University of Milan, Milan, Italy; 5 Stress, Psychiatry and Immunology Laboratory, Department of Psychological Medicine, Institute of Psychiatry, Psychology and Neuroscience, King’s College, London, UK

**Keywords:** Depression, Predictive markers

## Abstract

Depression and obesity represent two of the most common complications during pregnancy and are associated with severe health risks for both the mother and the child. Although several studies have analysed the individual effects of depression or obesity on the mothers and their children, the effects associated with the co-occurrence of both disorders have so far been poorly investigated. The relationship between depression and obesity is very complex and it is still unclear whether maternal depression leads to obesity or vice versa. It is well known that the intrauterine environment plays an important role in mediating the effects of both depression and obesity in the mother on the fetal programming, increasing the child’s risk to develop negative outcomes.

## Introduction

Depression and obesity are among the most prevalent complications during pregnancy and are associated with severe health risks for both the mother and her child. In the mother, the risks include gestational hypertension, pre-eclampsia, gestational diabetes, cesarean, and preterm delivery, a decreased breastfeeding initiation and duration, and a poor bonding of the mother with her child [1]. The association between depression and obesity is very complex as they influence each other. Although a few studies have reported no significant associations between maternal overweight and/or obesity and depressive symptoms or vice versa [2, 3], several lines of evidence have shown that among pregnant women, those with depression are more likely to develop obesity as compared with those nondepressed [4, 5]. Similarly, it has also been demonstrated that pregnant obese women are more vulnerable to develop depressive symptoms during pregnancy and/or in the postpartum period than normal weight, pregnant women are [6–9].

Depression and obesity are well-known risk factors for morbidity and mortality, but the extent to which these two conditions are causally related remains to be clarified [10]. Although most of the studies have focused the attention only on their individual effect, the co-occurrence of both disorders during pregnancy may lead to an even greater risk in the women and, as a consequence, their children [11]. Indeed, several lines of evidence have suggested that maternal depression and obesity can affect the next generation by increasing the child’s risk of preterm birth and lower birth weight, of suboptimal physical, cognitive and socio-emotional development, of poorer academic performance, and of physical and mental disorders in later life [1, 12–15].

Despite the intuitive conclusion that many pregnant women can experience both depression and overweight/obesity and that the combined exposure to these disorders is associated with an even greater risk of negative pregnancy outcomes, their dual effects have so far been poorly studied, but several biological and psychological pathways have been suggested to be involved. In particular, the hypothalamic-pituitary adrenal (HPA) axis deregulation, the inflammatory/immune system, metabolic dysfunctions, such as insulin resistance, and gut microbiota alterations have been proposed as biological mediators of this association, whereas body dissatisfaction, low self-esteem, stigmatization experiences, eating disorder psychopathology, as well as physical pain have been suggested as psychosocial mediators [16].

Against this background, in this review we aim to discuss data about the effects of depression, obesity, and their comorbidity during pregnancy on the mother’s health and on the offspring’s negative outcomes. We will discuss how different biological mechanisms altered in the fetal environment and associated with comorbid condition in pregnancy, may contribute in enhancing the risk for mental and metabolic disorders in the offspring’s lifespan. The identification of causative mechanisms and of putative targetable biomarkers that, in comorbid obese and depressed women, are associated with the offspring’s vulnerability, can allow the introduction of novel, timely and targeted intervention strategies starting already in early pregnancy, which may benefit the mother’s health as well as the child’s health and neurodevelopment.

## The fetal programming, the intrauterine environment and the effects on neonatal outcomes: role of stress and depression

Growing lines of evidence support an important role for the intrauterine environment in shaping the fetal development and the subsequent child’s health or disease risk. Indeed, the developing fetal brain is particularly plastic and sensitive to several in utero environmental adversities that can lead to long-term implications [17]. This phenomenon, known as “*early life programming,*” has been extensively studied in relation to individual exposure to maternal depression and/or obesity during pregnancy, but there is a lack of data about the effects of their comorbidity on the fetal development. In particular, a major knowledge gap concerns the (neuro)biological pathways that are altered by comorbid depression and obesity in pregnancy, and that shape the fetal vulnerability of developing altered mood and/or metabolic dysfunctions later in life.

**Fig. 1:**
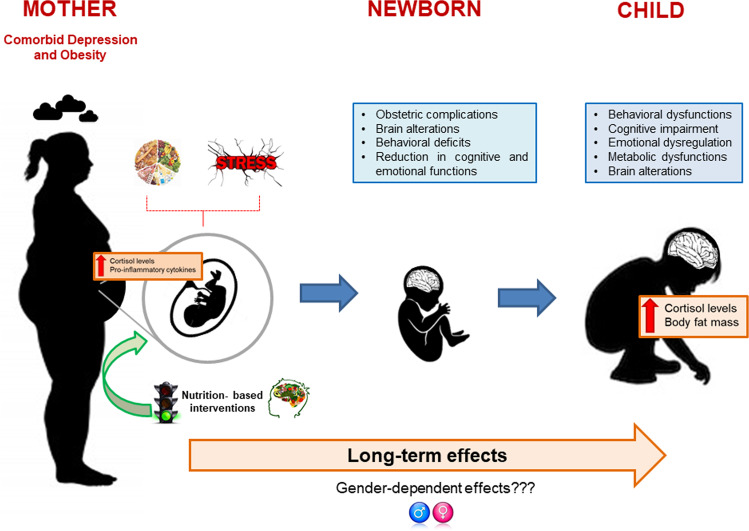
Effects in the transmission of vulnerability from comorbid depressed and obese mothers to their offspring. Maternal stress and nutrition can lead to alterations in the fetal programming, especially affecting the brain and increasing the risk for the offspring’s negative outcomes. Nutrition-based interventions can counteract these negative effects.

In this context, maternal stress represents a key factor that has received most of the attention, as it is known to influence the fetal development determining also long-term consequences (see Fig. 1). Interestingly, psychosocial stress exposure in pregnancy has been suggested influencing the infant temperament [18].

In the following paragraphs, we will describe the relationship between maternal stress and depression in pregnancy and their effects on the offspring’s behavioral and cognitive deficits; we will also discuss possible biological mechanisms underlying this association.

### Maternal stress

Fetal neuron proliferation, differentiation, migration, and aggregation, occurring for the entire gestational period, are biological processes that are mainly genetically determined and epigenetically directed; however, they are also strongly influenced by environmental factors [19], with maternal stress being one example of external adversities. Of note, the developing brain is the highest sensitive organ to the effects of stress in the prenatal period, as it undergoes substantial changes in structural growth and connectivity during the fetal life.

Several lines of evidence from preclinical models indicate the presence of long-lasting consequences of prenatal stress exposure, which include alterations both at behavioral and molecular level. For example, several paradigms of prenatal stress (i.e. restraint stress, forced swim stress, overcrowding, exposure to bright light and food deprivation) have been associated with the development of inhibited behaviors in the offspring, such as a reduced activity in the open field test, a reduced social preference, and an enhanced anxiety-like behavior in the elevated plus maze and light–dark box [20–22]. Moreover, independently from the type of the specific stressor applied, all these studies have consistently shown that prolonged prenatal manipulations impair spatial learning and memory, with an effect that can be observed not only in adolescence, but also in adulthood [23–26].

Epidemiological studies have also provided support to the preclinical data, showing for example that maternal stress during pregnancy, including exposure to bereavement, natural disasters or terrorism as well as financial and relationship problems, is associated with an increased incidence of difficult temperament and behavioral problems in the offspring, both during infancy and childhood [27–29]. In a prospective longitudinal study performed by Buss and collaborators, the effects of prenatal maternal stress and anxiety exposures on the offspring’s brain morphology have been assessed. Pregnancy stress and anxiety data were available at 19, 25, and 31 weeks of gestation, but the offspring were evaluated between 6 and 9 years of age by a structural Magnetic Resonance Imaging (MRI) scan. The results revealed changes in the brain morphology in children of mothers with anxiety-related symptoms during pregnancy. Specifically, anxiety at 19 weeks of pregnancy, but not at 25 and 31 weeks of gestation, was associated with gray matter volume reductions in several brain areas, including the prefrontal cortical and the medial temporal regions [30]. These findings suggest that these regions are shaped by and are the most sensitive brain areas in the offspring exposed to maternal stress and anxiety in utero. Similarly, in another interesting study, DiPietro et al. found that maternal psychological stress in pregnancy was associated with accelerated fetal neurological maturation, examined through the assessment of tone, posture, primitive reflexes, behavior and the measurement of brainstem auditory evoked potential, suggesting that the human brain requires sufficient but not excessive stress to promote neural development both before and after birth [31].

According to several lines of evidence, exposures to prenatal maternal stress occurring during the gestational period have sex-dependent effects on brain development within highly sexually dimorphic regions that are involved in the regulation of mood, stress responses, metabolic function, the autonomic nervous system, and the vasculature [5]. However, although the literature suggests that the sex-dependent impact of prenatal maternal stress is timing and brain region-specific, little is known about the mechanisms underlying these gender differences and the different vulnerability for the development of negative outcomes in male and female offspring.

Among all the brain regions, amygdala and hippocampus are those that have received particular attention in the context of the fetal developmental programming and stress, because their development starts at an early embryonic stage and they are believed to be particularly sensitive to elevated levels of glucocorticoids, the stress hormones, such as cortisol [32–34]. In this context, Buss et al. examined prospectively the association between maternal cortisol concentrations over the course of gestation with several measurements of the child hippocampus and amygdala volumes and other child clinical features related to affective functions. The authors found that higher maternal cortisol concentrations during early gestation were associated with larger right amygdala volumes, but specifically in girls at 7 years of age. The effect was still significant even after controlling for potential confounding effects, including maternal depression. The results also demonstrated an effect of higher maternal cortisol levels in early gestation on affective problems in girls and suggested that the presence of larger right amygdala volumes associated with higher cortisol levels may represent a mediator of the observed altered behaviors. In addition, these data supported that maternal cortisol can have a more powerful effect during an early phase of gestation, moderated by fetal/child sex [32], suggesting that the intrauterine environment may interact with fetal sex, in shaping the vulnerability of developing altered behaviors.

All these findings clearly indicate that early life stress exposure exert a key role on the fetal developmental programming, determining both short and long-term consequences on the exposed offspring, and suggest that interventions designed to shape the biological alterations associated with such exposures could plausibly have profound protective effects on fetal development and long-term health [35].

#### The role of glucocorticoids

The molecular mechanisms underlying the effects of obesity and depression during pregnancy on the intrauterine environment and on the fetus are still largely unknown; however several evidences now support the presence of alterations in pregnant obese and depressed women in the functioning of the HPA axis system, leading to an altered release of stress hormones, the glucocorticoids.

It is well known that glucocorticoids and their receptors (GRs) play a crucial role during the intrauterine development, driving both cellular growth and differentiation. During the last years, research has focused the attention on the effects of stress and depression during pregnancy on the regulation of the GRs and on the transmission of vulnerability to the offspring. It has been reported that fetal exposure to maternal depressed/anxious mood in the third trimester of pregnancy is associated with increased methylation levels of the glucocorticoid receptor gene (GR or NR3C1) in newborns and with increased salivary cortisol stress responses at 3 months [36]. Other studies have also reported that the expression levels of genes involved in GR functionality, such as genes encoding for the FK506 Binding Protein 5 (FKBP5), BCL2 Associated Athanogene 1, Nuclear Receptor Coactivator 1, and Peptidylprolyl Isomerase D are higher in association with a physiological pregnancy. Conversely, such upregulation is not observed in women suffering from depression in pregnancy, suggesting that GR-related chaperone gene expression levels over pregnancy may serve as a marker of an abnormal GR sensitivity in women at risk for depressive symptoms [37].

Another interesting study has associated the presence of higher maternal distress (perceived stress, depression, anxiety, and pregnancy-specific stress) with increased placental DNA methylation of FKBP5, which in turn predicted reduced fetal coupling [38]. Similarly, another report has described an association between higher placental DNA methylation of FKBP5 with increased arousal in newborns [39]. Since the presence of high levels of FKBP5 decreases the binding of cortisol to its receptor, leading to reduced cortisol responses, it has been suggested that increased FKBP5 DNA methylation may cause a more pronounced cortisol activation of GR target genes within the placenta, leading to an over activation of the stress response system also in the developing fetus [39].

Interestingly, Wang et al. found that genetic variants within FKBP5 gene in the offspring mediate the association between exposures to antenatal maternal depressive symptoms and the right hippocampal volume, with a trend for such moderation on amygdala volumes and cortical thickness. These findings suggest that the exposure to maternal depressive symptoms and the in utero development of specific brain regions can be moderated by genetic variants within the FKBP5 gene and that the transmission of the risk for depression from mothers to offspring is also mediated by the neonatal FKBP5 genotype [40].

Evidence from animals also suggests that exposure to inappropriate concentrations of glucocorticoids during sensitive neurodevelopmental periods can also increase the risk of developing metabolic disorders later in life. Several paradigms of stress in rodents including restraint stress and chronic mild stress in pregnant dams during the third week of gestation have indeed found that their offspring were heavier and exhibited greater adiposity, impaired glycemic control, and increased food intake when compared with unstressed controls [41–44]. In humans, the exposure to stress over pregnancy including maternal depression, maternal bereavement, or disaster-related maternal stress during pregnancy is associated with an increased risk, in the offspring, to develop metabolic disorders including obesity, type 2 diabetes, increased percent body fat, insulin resistance, and an unfavorable lipid profile [45]. In this regard, Entringer and collaborators examined, in a prospective longitudinal study, the association between maternal saliva cortisol production in early, mid, and late gestation with changes in infant body composition (percent fat mass) at birth and at 6 months of age. The authors found that maternal cortisol production in the later stage of pregnancy was positively associated with the magnitude of infant percent body fat change over the first 6 months of postnatal life, suggesting that exposure to high levels of glucocorticoids during fetal development may promote adipogenesis in the offspring with an effect already present at 6 months of age [46].

In another interesting study, Kumpulainen and collaborators studied whether maternal obesity early in pregnancy (before the 12th gestational week) was associated with diurnal salivary cortisol, a marker of HPA-axis activity, in young adult offspring. The results showed that offspring of mothers with high body mass index (BMI) had lower average levels of diurnal cortisol, especially in the morning, suggesting a prenatal programming, exerted by maternal obesity, of the offspring HPA-axis activity [47].

Dysregulation of the HPA axis activity in obesity has been also supported by other studies. Indeed, emerging literature data on the role of FKBP5 in metabolism regulation and obesity-related conditions have been reported [48]. Accordingly, glucocorticoids can induce FKBP5 expression in the adipose tissue, where higher FKBP5 expression has been associated with markers of insulin resistance, subcutaneous adipocyte diameter, and lower levels of plasma (high-density lipoprotein) HDL-cholesterol [49]. Moreover, FKBP5 genetic variants have been associated with type 2 diabetes, insulin resistance, and elevated serum triglycerides [50]. Similarly, in mice, Fkbp5 mRNA levels were found modulated by a high-fat diet and also by chronic social defeat stress in the hypothalamus and hippocampus, respectively, and hypothalamic Fkbp5 expression was found also associated with an increased body weight [51]. However, to our knowledge, no studies showing the association between FKBP5 and obesity during pregnancy neither in human cohorts nor in the pre-clinical models are available.

### Maternal depression

Depression affects about 6% of the adult population worldwide each year [52] and occurs about twice as often in women. It has been estimated that one in four women suffers from this disorder at some point during their lifetime [52–54] and, therefore, this involves pregnant women as well. Maternal depression during and immediately after pregnancy is indeed one of the most common perinatal complications and represents a critical public health problem, due to its prevalence and disability. It affects 10–15% of new mothers in both pregnancy and the postpartum period and it is associated with symptoms such as pervasive sadness, fatigue, and rumination, which could impact the mothers’ ability to bond with their baby in utero or postnatally. In turn, this may lead to a lack of mothers’ responsiveness to infant cues and to a disruption in the mother-child relationship [55]. Among the psycho-social-environmental risk factors, unwanted child, emergency cesarean section, marital conflict, having an infant with special needs or a lack of social/family support [56], poverty, lower social class and lower maternal education may promote the development of depressive symptoms in the postpartum period.

Nevertheless, all too often, depression is not diagnosed properly during pregnancy as the symptoms can be confused with hormonal imbalance [57]. Women who suffer from depression in pregnancy show often depressive symptoms also in the postpartum period [58]. Depression in pregnancy has been associated with children’s poor cognitive functioning, behavioral inhibition, emotional maladjustment, externalizing disorders as well as psychiatric or physical diseases later in life [59]. Indeed, the consequence of an exposure to maternal depression in utero to the offspring are not restricted to infancy, but their effects can be observed also at toddlerhood, adolescence, and adulthood. Similarly, it has been demonstrated that depression during the postpartum period leads to poorer mother-infant bonding, infant emotional variability already at 4 months of age [60] and to a range of several detrimental outcomes in infants and children [61], including a poorer infant motor development by 15 months [62], stunted growth at 2 years [63], and higher rates of morbidity (when monitored for the first 4 years) due to gastrointestinal and lower respiratory tract infections [64]. In addition, postpartum depression has been associated with poorer cardiac function in children at 9.5 years of age [65], with poorer child behavioral outcomes and cognitive abilities and with poorer language and intelligence development, as measured by intelligence quotient (IQ), throughout childhood [66].

Despite these common effects between depression in pregnancy and in the postpartum, we also need to underlie the presence of consequences which are associated specifically with depression in pregnancy or in the postpartum period. In fact, depression in pregnancy implies a biological physical relationship between the mother and the fetus, whereas the postpartum period is more characterized by a psychological relationship between the mother and her baby and it has been shown that depressive symptoms during this period cause mother’s carelessness and lack of care, which in turn affect bonding difficulties and insecure attachments.

According to several studies, depression in pregnancy causes alterations in several biological systems. For example, maternal depression in pregnancy is associated with higher cortisol levels, a proportion of which is likely to be transported via the placenta to the fetus. Placenta plays indeed a key role in mediating the maternal hormonal signals to the fetus and provides a barrier to fetal exposure to glucocorticoids levels in the maternal circulation [67] via the action of the 11-beta-hydroxysteroid dehydrogenase type 2 (11β-HSD-2) enzyme, which converts cortisol into its inactive form cortisone. However, several studies have shown that the presence of conditions, including stress and depression may reduce the expression of 11β-HSD-2 [68, 69], potentially exposing the fetus to deleterious high cortisol levels. Indeed, a low activity of placental 11β-HSD-2 has been associated with lower fetal weight and with higher stress reactivity in the offspring [68]. In line with these findings, recent data in pre-clinical models have also shown that placental deficiency of 11β-HSD-2 is associated with increased serotonin synthesis and impairments in the breakdown of this key neurotransmitter in the brain of the offspring [70]. Besides the key role of glucocorticoids, the condition of depression in pregnancy is also associated with a proinflammatory status, which can affect the developing fetus leading to negative effects on behavior and cognitive functions later in life [71, 72]. Indeed, cytokines have important effects on the fetal brain development, because they are involved in controlling neurodevelopmental trajectories, including temporal regulation of neurogenesis and gliogenesis, progenitor migration, proliferation, and axon path-finding, neuronal survival and synapse modulation and elimination [73, 74]. The physiological trajectory of fetal brain development thus requires a specific balance between pro- and anti- inflammatory constitutively expressed cytokines in the maternal and fetal environment. This balance is normally tightly controlled, but, in case of environmental insults, such as stressful experience or maternal depression, the excessively produced maternal proinflammatory cytokines can cross the placental boundary, reach the fetal compartments and stimulate the de novo synthesis of cytokines in the fetal brain [75].

Postpartum is a period that per se is characterized by sleep disturbances and heightened parenting stress. Caring of a newborn implies changes in the mother’s daily habits, reductions in personal time and may introduce new challenges, increasing the risk for depressed mood [76]. Although the mother and the newborn are not biologically bonded with each other, as in the womb, postpartum depression mainly affects the mother’s attachment with her child. On these bases, in a longitudinal birth cohort study Zou et al. [77] examined the effects of exposure to maternal depressive symptoms at different developmental stages from fetal life to preadolescence (10 years of age) on child brain development, examining volumetrics and white matter microstructure. The results showed that maternal depressive symptoms manifested when the child was 2 months old and were mainly associated with alterations in child brain development, suggesting that this early postnatal life period represents a critical window for brain development. In line with these data, a growing body of evidence suggests that during the postnatal period, the brain undergoes rapid growth in gray matter, mainly as a result of synaptogenesis and dendritic arborization [78]. Moreover, the postnatal period corresponds to the timing when the primary myelination process in white matter occurs [79], corroborating the hypothesis that high maternal depressive symptoms across the perinatal period may shape brain trajectories in the exposed children.

Studies in animal models and in humans have demonstrated that maternal anxiety and/or depression may alter the offspring’s brain anatomy and increase the risk of developing cognitive impairment and mental illnesses in the offspring, later in life [80, 81]. In addition, depression during pregnancy has been negatively associated with maternal-fetal attachment, such as the culmination of a woman’s own reflection on her pregnancy and motherhood, her enjoyment of pregnancy, excitement about mothering, and hopefulness for the future [82]. Indeed, an inverse relationship was found between the severity of depression and maternal-fetal attachment. This suggests that an improvement in depressive symptoms during pregnancy may potentially ameliorate maternal-fetal attachment and highlights the clinical importance of screening for depression during pregnancy, since it allows the early introduction of treatment.

#### Increased risk for the development of mental illnesses in the offspring of depressed mothers

Several studies have also shown that maternal depression during pregnancy predicts the offspring’s depression in adolescence and that child maltreatment represents a risk factor for the development of this disorder. Accordingly, by analyzing mother–offspring dyads of the South London Child Development Study, a prospective longitudinal birth cohort study set up by recruiting pregnant women in 1986, Plant and collaborators demonstrated that exposure to maternal depression during pregnancy increases the offspring’s vulnerability for depressive symptoms in adulthood. Specifically, the authors found that adult offspring exposed to maternal depression in pregnancy were 3.4 times more likely to have a DSM-IV depressive disorder, and 2.4 times more likely to have experienced child maltreatment, as compared with the nonexposed offspring [83]. Moreover, they found that the child maltreatment might represent a mediating mechanism linking exposure to maternal depression in pregnancy with the development of altered behaviors, including depression, during early adulthood.

Interestingly, Capron et al. compared the associations between maternal and paternal antenatal depression and anxiety during pregnancy with the offspring’s anxiety disorders, assessed by a structured psychiatric interview at 18 years of age, thus controlling for some genetic and similar environmental factors. Using data from the Avon Longitudinal Study of Parents and Children (ALSPAC Study), a large longitudinal birth cohort study based in and around Bristol (UK), the authors found an increase in the risk of anxiety disorders in adolescent offspring who were exposed to maternal depression in utero. Moreover, they observed that children of women with antenatal anxiety had increased risk of comorbid anxiety and depression later in life. No such associations were found when considering the exposure to paternal antenatal depression or anxiety [84], confirming, once again, that fetal programming is the main responsible biological mechanism for the association between in utero exposure to maternal antenatal depression and the offspring’s anxiety disorder. In another interesting study, Glynn et al. examined whether unpredictability of prenatal maternal mood, measured as an entropy score, might influence the developing fetus. The entropy score was calculated by applying Shannon’s entropy to the distribution of responses on four mood questionnaires individually administered at each visit. Briefly, participants who generally reported never worried or always secure on the State-Trait Anxiety Inventory (STAI) were considered very predictable and thus had a very low entropy score, whereas women who completed the STAI items entirely at random had a very high entropy score. The authors found that fetal exposure to more elevated maternal mood entropy predicted higher levels of child negative affectivity at 12, 24 months, and 7 years of age. In addition, children exposed to higher prenatal maternal mood entropy, reported higher levels of anxiety symptoms at 10 years and elevated depressive symptoms at 13 years [81]. Thus, according to the authors, mood entropy may not only represent a new target for intervention during the perinatal period, with the potential to increase treatments focused on depressive symptoms, but also a critical predictor of the mental health trajectories.

#### Increased risk for cognitive and emotional impairments in the offspring of depressed mothers

Several studies have also shown that maternal mood during pregnancy can predict alterations in child’s mental health and brain neurodevelopmental trajectories. On these bases, Raikkonen and collaborators have investigated whether maternal depressive symptoms, measured biweekly throughout pregnancy, twice up to 12 months postpartum, and when the child was 1.9–5.7 years old, were associated with child neurodevelopment in early childhood in a large prospective cohort of Finnish mothers and children (Prediction and Prevention of Pre-eclampsia and Intrauterine Growth Restriction study). The authors observed that children of mothers with the most chronic and severe depressive symptoms during pregnancy had the most neurodevelopmental disadvantages in terms of lower total score in fine and gross motor, communication, problem solving, and personal/social skills, and higher risk for total, internalizing and externalizing problems [85]. These findings suggest that the effects of depressive symptoms during and after pregnancy on child neurodevelopment are additive [86]. Moreover, these data implicate that interventions in early pregnancy may benefit both mothers and children, as an early treatment may prevent the accumulative cascade of depressive symptoms and may reflect positively on the child’s neurodevelopment.

An extensive meta-analysis performed by Goodman and collaborators to examine the strength of the relationship between mothers’ depression and children’s behavioral problems or emotional functioning has clearly indicated that depression in mothers is associated with children’s internalizing, externalizing problems and general psychopathology. Interestingly, when the authors took into account gender as a possible moderator, they showed that the association between maternal depressive symptoms and internalizing problems was stronger in girls than in boys. In contrast, this gender differences was not extended to externalizing problems or general psychopathology [87]. Thus, according to the authors, girls seem to be more sensitive to the postnatal stress context and to the style of parenting associated with depression in the mother. These findings raise interesting questions on how girls may be more vulnerable than boys to the development of internalizing problems when exposed to depression in the mother [87].

#### Increased risk for brain alterations in the offspring of depressed mothers

Maternal depression during pregnancy has also been associated with structural brain changes in child prefrontal cortex [88], frontal and inferior temporal areas [89], amygdala [90], and in neural connectivity between amygdala and fronto-striatal areas [91–93]. For example, in an interesting study, Sandman and collaborators examined the association between fetal exposure to maternal depression and cortical thickness in children 6–9 years old. Thus, a prospective, longitudinal study of maternal depressive symptoms at 19, 25, and 31 weeks of gestation was followed by the acquisition of a structural MRI scan in children. The results showed a significant cortical thinning primarily in the right frontal lobes in children exposed to prenatal maternal depression. Interestingly, the strongest association was observed at 25 weeks of gestation, suggesting that this gestational interval represents the most sensitive period for the fetus, resulting in broad cortical patterns of thinning. It is well known that the human fetus is responsive to rapid changes in maternal stress signals during midgestation, when axons form synapses with the cortical plate organizing cortical circuits (20–24 weeks) and when an intense neurons proliferation is occurring (about 40% greater than in the adult) (25–26 weeks). Thus, according to the authors, cortical thinning might constitute a brain-based endophenotype for the development of depression. These findings are in line with literature data showing cortical thinning and reduced activities in the frontal areas of adult depressed patients [88], and suggest that brain morphology alterations may occur early in life, already during fetal programming.

As the effects of depression in pregnancy appear to be gender-specific, Wen et al. examined the contributions of pre and postnatal maternal depressive symptoms on the volume and microstructure of the amygdala in 4.5 years old boys and girls. The results showed the presence of a larger right amygdala volume which was associated with greater prenatal maternal depression in girls, but not in boys, suggesting independent, differential influences of pre and postnatal depressive symptoms in the mother on the structural development of the amygdala, with evidence for gender-specific effects [33]. Moreover, these data emphasize the importance of the timing of exposure to maternal depression, as well as the importance of considering gender differences when planning intervention strategies for the prevention of adverse outcomes in exposed children.

#### Increased risk for the development of metabolic disorders in the offspring of depressed mothers

Evidence in rodents shows that alterations in the intrauterine environment, caused by exposures of the mother to stress during pregnancy may lead to alterations in the fetus development and in the baby at birth, such as a reduced weight [94]. These findings have been corroborated by several meta-analyses in human cohorts describing a small, but significant inverse association between stressful experiences in pregnancy and her child’s birth weight [95, 96]. Growing evidence has also suggested that depressive symptoms early in pregnancy are associated with metabolic dysfunctions in the mother. For example, it is well known that depression and type 2 diabetes mellitus interact bi-directionally during pregnancy, as one increases the risk of developing the other [97]. In this context, Ferrari et al. have investigated whether mild to moderate depressive symptoms in women after a recent pregnancy with a history of gestational diabetes were associated with pathologic glucose metabolism. Interestingly, in the group of women showing mild to moderate depressive symptoms, pathological glucose metabolism was more frequent and visceral fat and systolic blood pressure were higher than in the women without depressive symptoms (with and without adjustment for BMI). These data confirm the association between depressive symptoms during pregnancy, gestational diabetes, and an elevated risk for metabolic dysfunctions [98]. Similarly, several studies have suggested that depressive symptoms in the mother during pregnancy are associated with metabolic dysfunctions in the offspring. In this context, Silva and collaborators examined the associations of maternal overall psychological distress, depression, and anxiety during pregnancy with cardio-metabolic risk factors in 10-year-old children and explored potential sex-specific differences. In particular, they focused their attention on childhood blood pressure, heart rate, lipids profile, and glucose metabolism. Interestingly, maternal psychological distress in pregnancy was associated with higher childhood heart rate in boys, whereas maternal depression and anxiety correlated with higher childhood triglycerides only in girls. This suggests that the biological mechanisms underlying the association between maternal distress in pregnancy with metabolic alterations in children may be sex-specific [99].

## The fetal programming, the intrauterine environment and the effects on neonatal outcomes: role of nutrition and obesity

Maternal nutrition represents another risk factors known to influence the fetal development and to induce long-term consequences (see Fig. 1). In the following paragraphs, we will describe the relationship between maternal nutrition and obesity in pregnancy focusing our attention on their effects on the offspring’s behavioral and cognitive deficits.

### Maternal nutrition

During fetal development, adequate energy, and protein intake, essential fatty acids, and various key micronutrients (i.e. methionine, homocysteine, vitamins B6, B12, B9 (folic acid), and their metabolites) are required to supply the necessary substrates for the synthesis of fetal tissues and as cofactors in biochemical processes that coordinate the physiological brain development [100]. Thus, the diet of the future mother during the entire gestational period directly influences the fetus environment, which in turn can have an effect on the development of the fetal Central Nervous System (see Fig. 1).

Evidence from animal studies supports that offspring of undernourished pregnant dams show impaired neurogenesis and neuronal functionality, disorganization of feeding pathways, altered glucose sensing, and leptin and insulin resistance [17, 101]. Moreover, restriction in total calories or specifically macronutrients, such as low proteins, in pregnant dams has been associated with lower birth weight and several negative offspring’s outcomes [102]. Similarly, a high intake of fat or salt during pregnancy perturbs placental functions, alters fetal development, and predisposes the offspring to the development of several metabolic diseases in adult life. This was clearly demonstrated by Reynolds et al. who fed female Sprague-Dawley rats with a standard control diet, 4% salt diet, 45% fat diet or 4% salt/45% fat combined diet, 3 weeks prior to and throughout pregnancy and lactation. Plasma and tissue samples were collected from both mothers and fetuses at day 18 of pregnancy, a time point when the developing fetus was directly exposed to an altered maternal nutritional milieu, and at postnatal day 24 in weanlings. Pregnant dams who received high-fat and/or salt diets showed alterations in several markers associated with adipose tissue inflammation, macrophage infiltration, lipogenesis, nutrient transport, and storage. This was accompanied by an increased fat mass, differential hepatic lipid, and glucose homeostasis in the high-fat groups. Moreover, the offspring of high fat-fed mothers showed reduced fetal weight, displayed catch-up growth, increased fat mass, and altered metabolic profiles at weaning [103].

In humans, extreme cases of nutritional deprivation during pregnancy, such as in times of famine, have provided insights into the impact of malnutrition on the offspring’s brain development with a reduction in cognitive abilities [104, 105], neural tube defects [106] as well as language delay [107] as consequences. Similarly, a high protein maternal diet during late pregnancy is associated with an increased cortisol secretion in response to psychological stress in the offspring [108]. Consumption of high-fat diet during pregnancy not only leads to an excess gestational weight gain (GWG), but also worsens other maternal pregnancy outcomes such as dysglycemia, elevated blood pressure, blood lipid profiles, and risk of pre-eclampsia [109].

Interestingly, it has been reported that the diet of obese and depressed people is far from being adequate. They usually make poor food choices, selecting food that might actually contribute to obesity and/or depression. For example, the most common nutritional deficiencies seen in patients with mental disorders are of omega-3 fatty acids, B vitamins, minerals, and amino acids that are precursors of neurotransmitters [110].

Using a large longitudinal cohort, represented by the mother–offspring pairs participating in the Avon Longitudinal Study of Parents and Children (ALSPAC) in the United Kingdom, Barker et al. provided evidence that prenatal maternal depression is related to unhealthy prenatal diet, which, in turn, is prospectively associated with reduced cognitive functions in 8-year-old children [111]. Maternal depressive symptoms as well as maternal nutritional assessments were assessed at different time points during pregnancy and also after delivery. Maternal diet was divided into healthy and unhealthy diet; in particular, healthy foods included those high in proteins (i.e. fish, pulses), fibers (i.e. pulses) and in important nutrients such as folate, magnesium, potassium, and vitamins A, C and K (i.e. vegetables). Conversely, unhealthy foods were defined as being high in saturated fats, trans-fats (i.e. junk food), salt, and added sugar, which have been associated with obesity, poor health, and sedentary lifestyles. The authors found that higher maternal depressive symptoms were related to lower levels of healthy nutrition as well as higher levels of unhealthy nutrition, each of which was in turn prospectively associated with reduced cognitive functions in children [111]. These findings highlight an association between dietary habits in pregnancy and a reduction of cognitive functions in the child, suggesting the potential role for nutrition-based interventions in pregnancy to avoid cognitive deficits in the offspring.

Although dietary interventions have been shown to be an effective strategy for improving fetal and maternal outcomes in obese pregnant women [112], and a number of guidelines have been developed for the management of obesity in pregnancy [113], all of this has been infrequently translated into the clinical practice.

### Maternal obesity

During the last years, maternal obesity has become one of the most commonly occurring risk factors seen in the obstetric practice and the World Health Organization has described this trend as a “global epidemic burden” posing a serious threat to public health [114]. In 2016, over 650 million adults were obese, i.e. around 13% of the world adult population. At this time, over 340 million children and adolescents, aged 5–19 years were overweight or obese, with a prevalence of 18% in this age group. In this context, it is not surprising that rates of maternal obesity are also increasing [115]. Obesity is conventionally defined by the BMI, where BMI > 25 kg/m^2^ determines overweight, BMI >30 kg/m^2^ determines class-I obesity, BMI >35 kg/m^2^ determines class-II obesity and BMI >40 kg/m^2^ determines class-III obesity [116]. Although BMI is the commonly used method to determine and classify obesity, it does not always reflect the complexity of the biological picture. Indeed, it is questionable whether BMI should be used as the only index and parameter for obesity during pregnancy, given for example the gestational increase in water retention [117]. Moreover, many studies have supported the evidence that the outcomes observed in the offspring exposed in utero to maternal obesity are not only related to BMI itself, but to the effects that excessive pre-pregnancy weight has on the mother’s body, for example gestational diabetes mellitus, hypertension and pre-eclampsia [112]. Conversely, other studies have proposed that in some cases excessive GWG is more relevant than pre-pregnancy weight in shaping the offspring’s vulnerability [118, 119]. In particular, GWG is considered “excessive” when exceeding the recommended weight gain guidelines, which are: 12.5–18 kg for underweight women (BMI < 18.5), 11.5–16 kg for normal weight women (BMI = 18.5–24.9), 7.0–11.5 kg for overweight women (BMI = 25.0–29.9) and 5.0–9.0 kg for obese women [120]. Finally, as previously discussed, obesity is often accompanied by alterations in food intake, with preferences for junk food and with low vitamins and other fundamental nutrients intake, representing another possible pathway that could interfere with an optimal child’s brain and body development [121, 122].

#### Increased risk for the development of mental disorders in the offspring of obese mothers

Several studies from animal models have shown that a maternal diet high in fats disrupts the behavioral programming of the offspring, with newborn animals showing social impairments, increased anxiety and depressive behaviors, reduced cognitive development, and hyperactivity [1]. A recent meta-analysis on animal models indicates that the offspring of obese dams display higher levels of locomotor activity and anxiety behavior than the offspring of lean dams, but similar memory abilities [123].

Similar findings have been reported in literature showing that maternal obesity during pregnancy can affect the offspring’s brain development, increasing the risk of developing neurodevelopmental disorders, such as attention deficit hyperactivity disorder (ADHD), autism spectrum disorders, and schizophrenia [1, 12, 80, 124, 125]. In particular, it has been reported that children born from obese mothers or from mothers who gained excessive weight during pregnancy have a twofold increased risk of developing ADHD as compared with controls [119, 126–130].

In order to examine whether maternal obesity early in pregnancy could be associated with developmental delay in the offspring, a recent study by Girchenko et al. evaluated deficits in developmental outcomes in 23–69-months old offspring exposed to maternal obesity. Children have been tested by the mothers with the Ages and Stages Questionnaire (ASQ) third edition at a mean age of 42.1 months. The ASQ comprises 30 age-appropriate items and measures communication, gross motor, fine motor, problem solving, and personal-social skills. The authors evidenced that children of obese and overweight mothers had higher odds of failing to meet the developmental skills that are typical for that age. In particular, obesity during pregnancy was associated with delays in communication, fine and gross motor, problem solving and personal-social skills, whereas maternal early pregnancy overweight was associated only with delay in fine motor skills and mild delay in communication skills [131].

Other studies have suggested that maternal obesity and a high-fat diet consumption can enhance the offspring’s vulnerability for neurodevelopmental disorders by altering metabolic features, for example, by increasing maternal leptin, insulin, glucose, triglycerides, and inflammatory cytokines. Thus, based on this and in line with a recent review [12], the primary mechanisms possibly underlying the risk for neurodevelopmental disorders in the offspring of obese women include:

i. Oxidative stress and inflammation-induced mal-programming.
ii. Dysregulation of insulin, glucose, and leptin signaling in the developing brain.
iii. Dysregulation of dopaminergic and serotonergic signaling and impaired reward circuitry.
iv. Alterations in synaptic plasticity.

All together, these findings confirm the association between obesity in pregnancy and the enhanced risk for neurodevelopmental delays in the exposed offspring and emphasize the importance of optimizing weight management during pregnancy, since it is likely to shape the offspring’s vulnerability to develop behavioral and cognitive impairments.

#### Increased risk for the development of metabolic dysfunctions in the offspring of obese mothers

One of the first obvious effect of maternal obesity on the offspring’s health is the possibility of developing metabolic dysfunctions and gaining excess weight already in infancy and adolescence [132]. Indeed, maternal obesity can induce alterations on infants’ body composition already within the first years of life. For example, in a Swedish study, children of normal weight women (controls) and those of obese mothers with gestational diabetes have been checked at 1 week, 12 weeks, and 1 year of age. Weight, length, and BMI have been measured, along with body fat and evaluated with the air-displacement plethysmography, a recognized and scientifically validated densitometric method to measure the human body composition. The differences between controls and infants exposed to obesity in utero were already evident at 1 week of age: at this early age, indeed, obese mothers’ offspring displayed higher weight as compared with their controls. Interestingly, fat mass percentage was also higher at 1 and 12 weeks of age in the obese and gestational diabetes offspring groups, but only in girls. In particular, girls from obese mothers showed higher BMI at 12 weeks of age, whereas boys reached statistical significance at 1 year of age. Moreover, obese mothers’ children exhibited a more rapid growth than the normal weight group, whereas gestational diabetes mellitus did not correlate with infant weight at any time, suggesting again that maternal obesity, but not diabetes, could have an impact on children’s body composition even at early age [133].

Another interesting follow-up study has focused the attention on the association between multiple maternal metabolic parameters (pre-pregnancy BMI, GWG, gestational diabetes mellitus, non-fasting serum concentrations of cholesterol, triglycerides, and C-reactive protein (CRP)) and the offspring’s BMI trajectories during the first 4 years of postnatal life [118]. Children, who have been visited at birth, 6 months, 1, 2 and 4 years of age, were divided into five groups according to similar BMI *z* score trajectories, such as longitudinal BMI trajectories that integrate information on multiple aspects of growth (birth size, BMI gain velocity, blood pressure). These groups included children with high weight at birth and accelerated weight gain (class 1), high weight at birth and slow weight gain (class 2), low weight at birth and accelerated weight gain (class 3), normal weight at birth and slow weight gain (class 4, the reference group), low weight at birth and slow weight gain (class 5). Interestingly, pre-pregnancy obesity positively correlated with class 1 and 3, whereas excessive GWG positively correlated only with class 1, suggesting that both pre-pregnancy obesity and excessive GWG could accelerate BMI gain in the offspring from birth through early childhood [118]. Indeed, children of mothers who gained weight above the clinical recommendations, as compared with children of mothers within the recommended range, had a higher probability to follow a BMI trajectory of higher birth size and accelerated BMI gain [118]. Maternal cholesterol and CRP levels in early pregnancy were not associated with child’s BMI trajectories, whereas serum triglycerides correlated with longitudinal BMI trajectories in early childhood, thus explaining the increased disease risk later in life.

Another similar study has assessed children every year from birth until 4 years of age and, based on the results, children were divided into three BMI trajectories: rising-high BMI (group 1), moderate BMI (group 2), low BMI (group 3). Again, both pre-pregnancy obesity and excessive GWG were significantly associated with the rapid weight gain, whereas gestational diabetes mellitus was only associated with higher BMI at 4 years of age [134]. In another interesting work, Rasmussen et al. investigated whether the gray matter volume in the insula of newborns could be associated with alterations in the percentage of body fat accrual over the first 6 months of postnatal life, a key risk factor for childhood obesity. Indeed, insula is a brain area involved in somatosensation, interoception, gustation, olfaction, reward/addiction, and emotion and it has a relatively large structure that can be easily characterized in the newborn brain [135]. By conducting a prospective, longitudinal study in a population-based cohort of mother-infant dyads from gestation through birth until 6 months postnatal age and by performing MRI and whole body Dual-energy X-ray Absorptiometry imaging, the authors observed an inverse association between small insula gray matter volume and an increase in percent body fat at 6 months postnatal age, thus suggesting an underlying neural basis of childhood obesity and confirming again the key role of the intrauterine environment.

Several studies have also investigated the relationship between the mother and the offspring’s BMI, highlighting conjointly the influence of excessive GWG and/or gestational diabetes mellitus (Table 1). For example, one of the latest studies that have focused on the long-term effects of maternal obesity has examined: (i) the offspring of normoglicemic mothers with overweight/obesity, (ii) the offspring of mothers with gestational diabetes mellitus, both normal weight and overweight/obese, (iii) the offspring of normal weight and normoglicemic mothers (controls) at 20 years of age. The authors found that the offspring of normoglicemic obese mothers displayed higher BMI and body fat percentage, measured with bioimpedance analysis, a noninvasive, low cost and commonly used approach for body composition measurements and assessment of clinical conditions, as compared with controls. Similarly, the offspring of mothers with gestational diabetes mellitus showed an increased fat percentage, but when mother’s BMI was taken into account, the greatest differences in BMI fat deposition were observed between the offspring from obese mothers with diabetes and controls, whereas the offspring from lean mothers with gestational diabetes did not display such a great difference. These findings suggest that both BMI as well as gestational diabetes in pregnant women have an influence on the offspring’s BMI and fat deposition at 20 years of age, with the mother’s BMI showing the strongest effect [136].

**Table 1:** Most important studies that in the last 5 years (from 2014 to 2019) have investigated the relationship between the mother and the offspring’s BMI, taking into account the influence of excessive GWG and/or gestational diabetes mellitus.

Authors	Title	Journal	Year	Main findings
Gademan et al.	Maternal prepregancy BMI and lipid profile during early pregnancy are independently associated with offspring’s body composition at age 5–6 years: the ABCD study.	PLoS ONE	2014	Overweight and obese mothers and mothers with pronounced free fatty acids levels during early pregnancy were more likely to have children with overweight or obesity at age 5–6years. Both pre-pregnancy BMI and maternal lipids during early pregnancy are independently related to offspring adiposity.
Page et al.	Gestational diabetes mellitus, maternal obesity, and adiposity in offspring.	J Pediatr.	2014	Offspring of mothers with gestational diabetes mellitus had greater BMI and waist and hip circumferences compared with offspring of non-gestational diabetes mellitus mothers. However, this relationship is not mediated by maternal obesity.
Eriksson et al.	Maternal weight in pregnancy and offspring body composition in late adulthood: findings from the Helsinki Birth Cohort Study (HBCS).	Ann Med.	2015	Maternal BMI was positively associated with BMI in the offspring. A significant interaction between birth weight and maternal BMI on offspring body fat percentage was found.
Tan et al.	Mother’s pre-pregnancy BMI is an important determinant of adverse cardiometabolic risk in childhood.	Pediatr Diabetes	2015	Children of overweight or obese mothers at the time of conception have a greater risk to be overweight/obese with increased total body and abdominal adiposity compared with children born to normal weight mothers. They also manifest insulin resistance and an adverse metabolic profile in later childhood.
Leng et al.	GDM Women’s Pre-Pregnancy Overweight/Obesity and Gestational Weight Gain on Offspring Overweight Status.	PLoS One	2015	Pre-pregnancy overweight/obesity and excessive GWG of gestational diabetes mellitus mothers were positively associated with increased risks of childhood overweight of their offspring at 1–5 years old.
Hillier et al.	Impact of Maternal Glucose and Gestational Weight Gain on Child Obesity over the First Decade of Life in Normal Birth Weight Infants.	Matern Child Health J	2016	Both maternal hyperglycemia and excessive weight gain have independent effects to increase childhood obesity risk during the first decade of life.
Cosson et al.	Pregnancy adverse outcomes related to pregravid body mass index and gestational weight gain, according to the presence or not of gestational diabetes mellitus: A retrospective observational study	Diabetes Metab.	2016	Both overweight/obesity and GWG are crucial for fetal growth in women without gestational diabetes mellitus, with the role of BMI blunted in women treated for gestational diabetes mellitus.
Contreras et al.	Maternal pre-pregnancy and gestational diabetes, obesity, gestational weight gain, and risk of cancer in young children: a population-based study in California.	Cancer Causes Control.	2016	Children of mothers with pre-pregnancy diabetes have an increased risk for several childhood cancers, such as leukemia and Wilms’ tumor.
Ouyang et al.	Maternal BMI, gestational diabetes, and weight gain in relation to childhood obesity: The mediation effect of placental weight	Obesity	2016	Pre-pregnancy obesity, excessive GWG, and gestational diabetes mellitus were all associated with greater childhood BMI and higher risk of childhood obesity from infancy to7 years of age. Higher placental weight was an independent predictor of offspring BMI and obesity.
Maslova et al.	Maternal protein intake in pregnancy and offspring metabolic health at age 9–16 y: results from a Danish cohort of gestational diabetes mellitus pregnancies and controls.	Am J Clin Nutr.	2017	A modest, but not significant, increase in offspring abdominal adiposity was associated with a higher maternal protein intake in gestational diabetes mellitus-exposed offspring
Bider-Canfield et al.	Maternal obesity, gestational diabetes, breastfeeding, and childhood overweight at age 2 years.	Pediatr Obes.	2017	Maternal pre-pregnancy obesity and overweight had the greatest association with childhood overweight at age 2 years
Kaseva et al.	Pre-pregnancy overweight or obesity and gestational diabetes as predictors of body composition in offspring twenty years later: evidence from two birth cohort studies.	Int J Obes	2018	Maternal pre-pregnancy overweight and gestational diabetes mellitus are associated with unhealthy body size and composition in offspring over 20 years later.
Gomes et al.	Late-pregnancy dysglycemia in obese pregnancies after negative testing for gestational diabetes and risk of future childhood overweight: An interim analysis from a longitudinal mother-child cohort study.	PLoS Med.	2018	Offspring of obese mothers treated and monitored because of a diagnosis of gestational diabetes mellitus appeared to have a better BMI outcome in childhood than those of obese mothers who remained untreated in the last trimester and developed an abnormal glucose metabolism.
Hammoud et al.	Long-term BMI and growth profiles in offspring of women with gestational diabetes.	Diabetologia	2018	Offspring of mothers with gestational diabetes mellitus appear to be at particularly higher risk of being overweight in adolescence.
Ott et al.	Maternal overweight is not an independent risk factor for increased birth weight, leptin, and insulin in newborns of gestational diabetic women: observations from the prospective ‘EaCH’ cohort study.	BMC Pregnancy Childbirth.	2018	Neither overweight/obesity nor gestational weight gain appear to be independent determinants of increased birth weight, insulin, and leptin. However, 3rd trimester glucose values were positively associated with critical birth outcomes.
Wang et al.	Maternal Gestational Diabetes and Different Indicators of Childhood Obesity - A Large Study.	Endocr Connect.	2018	Children born to gestational diabetes mothers had higher Z-weight, Z-BMI, waist circumference, body fat, subscapular skinfold and suprailiac skinfold, and were associated with increased risks of overweight, obesity, and high body fat. These associations were independent of maternal prepregnancy BMI, gestational weight gain, and other related maternal and infant factors.
Kaseva et al.	Gestational Diabetes But Not Prepregnancy Overweight Predicts for Cardiometabolic Markers in Offspring Twenty Years Later.	J Clin Endocrinol Metab.	2019	Maternal gestational diabetes mellitus was associated with increased insulin resistance and the risk of an atherogenic lipid profile in the adult offspring. Maternal prepregnancy overweight/obesity was associated with impaired offspring glucose regulation.
Hildén et al.	Gestational diabetes and adiposity are independent risk factors for perinatal outcomes: a population based cohort study in Sweden.	Diabet Med.	2019	The impact of maternal excess weight on adverse perinatal outcomes did not differ significantly between the offspring of mothers with and without gestational diabetes mellitus. Maternal overweight and obesity and gestational diabetes mellitus are major, independent risk factors for most adverse perinatal outcomes.

#### Increased risk for the development of cardiovascular diseases in the offspring of obese mothers

The eventuality of obesity in the pregnant mother as well as in the offspring at early age might lead to other dangerous pathologies later in life, with an adverse profile already observable during childhood. In fact, it is well known that higher maternal BMI, a high pre-pregnancy weight or an excessive GWG are likely responsible for adverse cardiovascular risk factors in children between 6 and 10 years old. High mother’s BMI and GWG have been indeed associated with higher blood pressure, adverse lipid profile, insulin resistance and higher inflammatory markers such as Interleukin-6 and CRP in children, suggesting that in utero exposure to a nonhealthy metabolic environment provides the basis for cardiovascular diseases in the offspring [137–139].

To sum up, higher BMI and excessive GWG in pregnancy have been associated with several negative consequences in the offspring, such as neurodevelopmental deficits, metabolic dysfunctions, and cardiovascular diseases. Nevertheless, it is still unknown the causal relation between maternal obesity and the negative outcomes in children. In this context, experimental models provide a tool that allow the researchers to investigate on this topic, and the most commonly used method to induce obesity in animals is a nutrition based on high-fat diet and/or junk food [140]. Moreover, animal models allow investigations that are impossible on human subjects, such as the evaluation of different developmental stages in utero or the long-term effects on the offspring’s negative outcomes in brief periods [140].

## The combined effects of mental and metabolic adversities in pregnancy on neonatal outcomes

In this section, we will describe the comorbid relationship between maternal stress and nutrition but also between depression and obesity in pregnancy and their effects on the offspring’s negative outcomes. In particular, we will try to understand whether they act through similar mechanisms.

### Maternal stress and nutrition

Although most of the literature considers only the independent effect of exposures to maternal stress and malnutrition on the developing fetus, recent studies have focused the attention also on the copresence of both factors, also because they are interconnected with each other.

Indeed, extreme maternal diet influences maternal stress responses and similarly, maternal stress affects maternal feeding behavior. It is well known that stress induces a preference for a high-fat and high-sucrose diet, which can dampen the cortisol stress response, giving rise to a state of emotional eating, and to an increased susceptibility for weight gain and other metabolic dysfunctions. Moreover, the interaction between stress and high-fat diet consumption has been demonstrated to be able to induce a proinflammatory response in women, which in the context of pregnancy could promote alterations in the offspring’s brain structure and connectivity [17] through the exposure of the developing fetal brain to altered cytokines levels [75] (see Fig. 1).

Interestingly, Naninck and collaborators suggested that a peripheral and central restoration of the early life stress induced reduction in the methionine levels, an essential 1-carbon metabolism-associated micronutrient (1-CMAM), via supplementation of the dam’s diet during the early stress period, has long-lasting benefits on the offspring’s behavioral and cognitive related performances [141]. In fact, this short dietary intervention was able to counteract the negative effects of early life stress in the object recognition performance test on cognitive parameters and to improve spatial learning and memory in the Morris water maze tests. This indicates that the lack of methionine during this critical period is a determinant factor for the development of cognitive impairments and supports the notion that essential micronutrients implicated in 1-carbon metabolism can counteract the negative programming effects of early life stress on later cognitive function abilities. Furthermore, the authors found that a maternal diet enriched in 1-CMAMs prevents the hyperactivation of the HPA axis induced by early life stress. Similarly, Schulz et al. reported that dietary choline supplementation given to stressed dams during pregnancy and lactation ameliorated the anxiogenic effects induced by prenatal stress in the offspring. Moreover, choline exposure increased exploratory behavior only in females but not in male offspring, indicating that perinatal choline supplementation prevents, with a gender-specific effect, the consequences of prenatal stress and suggesting the importance of a nutrition-based intervention during pregnancy [142].

At the clinical level Brunst et al. have suggested that the effects of prenatal maternal stress on infant temperament can be modified in a race-dependent manner by nutrients, such as daily intakes of polyunsaturated fatty acids (PUFAs) (n3, n6). Specifically, African-American infants whose mothers were exposed to increased social stress and had lower n3:n6 ratio intakes in pregnancy showed alterations in the temperamental profiles, as shown by their lower infant Orienting & Regulation scores; conversely, higher n3:n6 ratio intakes appeared to ameliorate the effects of prenatal stress exposure [18]. These data suggest that an optimal PUFAs ratio may protect the fetus from the debilitating effects of stress, in a race specific manner, particularly among African-Americans. Similarly, Monk et al. highlighted that maternal consumption of certain nutrients (e.g. proteins, B vitamins, folate, fats, iron, zinc, and choline) not only has positive effects on brain development but can also influence the stress response. Indeed, whereas it is well known that maternal stress during pregnancy can decrease the postpartum iron status in infants [143], an additional intake of iron to the mother may counteract the negative effects of stress.

### Maternal depression and obesity

Although maternal overweight/obesity and depression have been individually associated with similar underlying mechanisms and adverse effects on the offspring, only a few studies have accounted for the presence of both these pregnancy complications and their contribution to the offspring’s mental and metabolic disorders. Recently, McDonald and collaborators have clearly indicated that the impact on the risk of adverse neonatal outcomes is even higher when both maternal obesity and maternal depression occur, suggesting that women with pre-pregnancy obesity and depression constitute a particular high-risk group [11]. Among the 70605 women enrolled in the study, more than half (50.3%) were overweight or obese according to their BMI; 5% of lean women reported being depressed during the current pregnancy or having a history of depression, as compared with 6.2% of overweight/obese women. Depression and excess pre-pregnancy weight were individually associated with an increased risk of adverse neonatal outcomes, such as stillbirth, neonatal death, preterm birth, birth weight <2500 g, admission to a neonatal special care unit, or a 5-min Apgar score <7, but the highest risk was seen when they were both present (16% of nondepressed healthy weight pregnant women, 19% of depressed healthy weight women, 20% of nondepressed overweight/obese women and 24% of depressed overweight/obese women). These higher risks of adverse neonatal outcomes persisted after accounting for potential confounding variables, such as maternal age, education, and pre-existing health problems.

Although the percentage of women experiencing comorbid obesity and depression during pregnancy is rising in the last years, no follow-up studies have been conducted to investigate the effects and the timing of both the comorbid disorders on the offspring’s health during their lifetime. Indeed, this would be of great help to understand the relationship between maternal obesity and depression during pregnancy and to identify the altered molecular mechanisms and biological targets that play a key role in the transmission of the disease vulnerability across generations. Interestingly, the state of the art suggests that, on one hand, pre-pregnancy obesity, an excess GWG and a high-fat diet facilitate depressive and anxiety-like behaviors in the offspring [144, 145], whereas, on the other hand, high maternal stress and depression during pregnancy are related to maternal weight gain, worst birth outcomes and health trajectories for women and infants [146]. Indeed, a recent study has shown that depressed pregnant women have significantly higher heart rates, lower heart rate variability, and give birth to heavier babies than those of nondepressed pregnant women [146].

To address these critical gaps in the literature, Kumpulainen et al. have recently tested in a large sample of pregnant Finnish women whether BMI in early pregnancy could be associated with depressive symptoms reported by the mother biweekly, from the 12th until the 39th gestational week or delivery, and twice at about 2 and 28 weeks after delivery. The authors found that maternal early pregnancy overweight and obesity are associated with consistently higher levels of depressive symptoms throughout gestation, confirming the strong association between obesity and depression during and after pregnancy [147]. Similarly, Cunningham et al. have shown that adolescents with excessive GWG who enter pregnancy overweight or obese have significantly higher postpartum depressive symptoms as compared with those subjects with healthy pre-pregnancy BMI and appropriate GWG [148].

The relationship between depression and obesity during pregnancy was clearly demonstrated by Laraia and collaborators who tested the feasibility of two novel intervention programs aimed at reducing stress and overeating in the meantime during pregnancy [149]. Indeed, the authors adapted a mindfulness-based intervention that focused on the acceptance of negative experiences to promote self-regulation (Mindful Moms Training), and an active coping intervention that focused on reappraisal to promote intentional change in the levels of stress (Emotional Brain Training). Interestingly, after 8 weeks both interventions promoted meaningful reductions in stress and depressive symptoms and improved eating behaviors in a high-risk group of low-income pregnant women [149].

These findings confirm the strong interaction between depression and obesity during pregnancy, especially in the mother, but so far, no studies have taken into account the long-term effects of this comorbid condition on the offspring.

## Conclusions

In this review, we have discussed the relationship between depression and obesity during pregnancy, which affects both the mother and the offspring’s mental and physical health. Indeed, this comorbid condition increases the risk for adverse neonatal outcomes and may enhance the long-term vulnerability for several mental disorders, metabolic dysfunctions, and cardiovascular diseases in the offspring.

Interestingly, we have also confirmed that the intrauterine environment, with maternal stress and altered nutrition representing the two most relevant affecting factors, plays a key role in shaping and driving the fetal programming and the offspring’s vulnerability. We have discussed the possible biological causes associated with the different vulnerability between male and female offspring exposed to depression and/or obesity during the fetal life; however, as only a few studies have focused on gender differences, further analyses are needed to understand this. Moreover, to our knowledge, no follow-up studies have analysed the effects and the timing of both comorbid depression and obesity on the offspring’s negative outcomes. This kind of studies would be of great help, as they would allow the identification of the underlying deregulated biological mechanisms and the putative biomarkers for an early detection of high-risk pregnant women and their children that could thus benefit from preventive interventions.

Literature data about the safety and efficacy of antidepressants during pregnancy have not been able to demonstrate the lack of their safety or the clear presence of negative consequences on the offspring associated with the administration of antidepressants in pregnancy. However, non-pharmacological therapies, such as nutrition-based interventions and psychotherapy have been recently demonstrated as a strong adjuvant tool for the treatment of maternal depression in pregnancy and for the prevention of negative outcomes in children. For example, omega-3 PUFAs may have therapeutic benefits in depression during pregnancy since a profound decrease of omega-3 PUFAs in the mother during pregnancy is associated with a higher demand of fetal development and might precipitate depressive symptoms [150]. Indeed, Nishi and collaborators have recently conducted a double-blind randomized clinical trial called SYNCHRO (Synchronized Trial on Expectant Mothers with Depressive Symptoms by Omega-3 PUFAs) and they have established that depressed pregnant women receiving a moderate dose of omega-3 PUFAs (1.8 g consisting of 1.2 g of eicosapentaenoic acid (EPA) and 0.6 g of docosahexaenoic acid (DHA) daily) experienced great improvement in depressive symptoms as those receiving placebo [151]. Similarly, in another interesting study Hsu et al. have shown that supplementation with EPA-rich oil is able to effectively reduce depression during pregnancy as well as postpartum depression after childbirth. Moreover, long-term treatment with DHA-rich oil, an important element for the neuron cell membrane, can be effective in reducing the risk of postpartum depression in healthy women [152].

Interestingly, in a recent review, Middleton et al. [153] have suggested that omega-3 PUFAs administration during pregnancy is also associated with an improvement of negative outcomes and obstetric complications in children. Indeed, they found that the incidence of preterm birth (before 37 weeks) and very preterm birth (before 34 weeks) was lower in women who received omega-3 PUFAs as compared with those that did not receive omega-3. The risk of the baby to die or to be very sick and to require neonatal intensive care was lower in the omega-3 PUFAs group. However, despite all these findings, further follow-up studies are needed to assess long-term outcomes for both mothers and their children, and to establish if and how the outcomes can be influenced by the type of omega-3 PUFA, by the timing and doses, or by socio-demographic characteristics of women [153]. Moreover, above to nutrition-based interventions, also psychotherapy, mainly based on mindfulness techniques and exercises, have been associated with improved levels of general and pregnancy-related mood in pregnant women [154].

In conclusions, data discussed in our review suggest that depressed and obese women during pregnancy, which is per se an important and sensitive period for a woman’s life, represent a particular high-risk group that need more attention and more public initiatives to improve their mental and physical health and those of their offspring.
